# Comparison of Time to Intubation of a Double-Lumen Endobronchial Tube Utilizing C-MAC® Versus GlideScope® Versus Macintosh Blade: A Randomized Crossover Manikin Study

**DOI:** 10.7759/cureus.50523

**Published:** 2023-12-14

**Authors:** Srinivasan Rajagopal, Richard N Gardner, Elizabeth Swanson, Sung Kim, Rakesh Sondekoppam, Kenichi Ueda, Satoshi Hanada

**Affiliations:** 1 Anesthesia, University of Iowa Hospitals and Clinics, Iowa City, USA; 2 Tippie College of Business, University of Iowa, Iowa City, USA; 3 Anesthesia, Kameda Medical Center, Kamogawa, JPN

**Keywords:** laryngoscopy, macintosh, glidescope, c-mac, double lumen endobronchial tube

## Abstract

Background: Macintosh blade direct laryngoscopy is widely used for endotracheal intubation. It may, however, provide an incomplete view of the glottis in patients with challenging airway anatomy. Consequently, various video laryngoscopes have been developed to enhance the visualization of the glottis and facilitate intubation. Yet, the effectiveness of these video laryngoscopes for intubation using a double-lumen endotracheal tube (DLT), which is longer, larger, and more rigid and has a linear configuration as opposed to the naturally semicircular curvature of a single-lumen endotracheal tube, remains uncertain. We hypothesized that video laryngoscopes would be more efficient for DLT intubation compared to the Macintosh blade in an adult manikin.

Methods: Ninety-four anesthesia providers, comprising 67 residents, 15 fellows, and 12 attendings, attempted to intubate an adult manikin with normal airway anatomy (Laerdal, Wappingers Falls, NY, USA) using a 37 Fr left-sided DLT. Three different intubation devices were used: the C-MAC® video laryngoscope (Karl Storz GmbH & Co. KG, Tuttlingen, Germany), the GlideScope® video laryngoscope (Verathon Inc., Bothell, WA), and the Macintosh blade direct laryngoscope-were used. Each participant intubated a manikin once with each of the three devices. Participants were randomized via a crossover design with the order of devices determined by using a Latin square design. Time to intubation and the number of failed intubations (esophageal intubation) were compared across the three different devices.

Results: Mean times to intubation for the C-MAC®, GlideScope®, and Macintosh blades were 18.57 ± 0.77, 36.26 ± 2.69, and 20.76 ± 0.96 seconds, respectively. There was a statistically significant difference (P<0.001) between the GlideScope® and the other two laryngoscopes. The times for C-MAC® and Macintosh blades were not significantly different. There were two instances of first-attempt failed intubation with the Macintosh.

Conclusion: Both the C-MAC® and the Macintosh blades proved more efficient in terms of time to DLT intubation in the manikin with normal airway anatomy, when compared to the GlideScope®. Considering the occurrence of first-attempt failed intubation, the C-MAC® was the most effective device among the three laryngoscopes for timely successful DLT intubation in the adult manikin. Further studies are needed to confirm these results in human subjects.

## Introduction

Thoracic procedures involving one-lung ventilation (OLV) present a unique challenge to anesthesiologists. While OLV improves access to the operation field and expedites the surgical process, anesthesiologists must ensure adequate ventilation and oxygenation. To accomplish OLV, anesthesiologists often use a double-lumen endotracheal tube (DLT) [1]. Compared to standard single-lumen endotracheal tubes, the DLT has a larger diameter, a longer length, and a more rigid structure. These factors make intubation with DLT much more difficult in both normal and difficult airways, which could increase the overall morbidity and mortality risk to the patient due to upper airway trauma and post-operative complications [2-5]. Moreover, DLT use is contraindicated in cases involving a difficult airway, severe tracheal stenosis, or significant distortion of the airway anatomy, all of which may preclude the use of DLT.

Macintosh blade direct laryngoscopy has been widely used to place both a single-lumen endotracheal tube and DLT. However, an incomplete view of the glottis during direct laryngoscopy due to the challenging airway anatomy often increases the difficulty and duration of achieving endotracheal intubation, hence increasing the risks of hypoxia and trauma to the patient [5,6]. To address the need for easier and safer endotracheal intubation, several different types of video laryngoscopes have been developed. The C-MAC® (Karl Storz GmbH & Co. KG, Tuttlingen, Germany) is a video laryngoscope, and its standard blade has an identical shape to the Macintosh blade; however, its camera attachment allows for both direct and indirect views of the glottis. The C-MAC® has demonstrated higher success rates of tracheal intubation compared with direct laryngoscopy in difficult airway patients [7-9]. GlideScope® (Verathon Inc., Bothell, WA, USA) is another video laryngoscope in which the glottis is indirectly viewed through an optical video apparatus embedded in a 60-degree angulated blade. Studies have also shown that the GlideScope® is more effective than direct laryngoscopes in difficult airway intubations with standard single-lumen tubes by producing a faster intubation time, a higher success rate, and less tissue damage [10,11]. Nevertheless, the effectiveness of these devices when placing a DLT remains unclear. Therefore, this study aims to compare the efficiency of DLT intubation using two different video laryngoscopes, C-MAC® and GlideScope®, and a traditional Macintosh blade direct laryngoscope in a manikin. We utilized a manikin to ensure a standardized comparison and to eliminate patient-related variables, which also enhances safety by avoiding potential airway trauma. We hypothesize that video laryngoscopes will facilitate more efficient intubation compared to the Macintosh blade due to better glottic visualization. Additionally, this study aims to assess if the level of training and experience of the anesthesia provider impacts the efficiency of DLT intubation across these devices. The primary outcome measured is the time to successful intubation, and the secondary outcome is the incidence of failed intubations, specifically esophageal intubations. 

## Materials and methods

This is a randomized, crossover manikin study approved by the Institutional Review Board at the University of Iowa (IRB # 201211726). Written consent was obtained from all participants prior to enrollment in the study. Participants were recruited from the University of Iowa directory. A preapproved email was sent to interns, residents, fellows, and attending physicians in the Department of Anesthesia at the University of Iowa Hospitals & Clinics. The inclusion criteria were licensed physicians who worked at the University of Iowa and held either a Doctor of Medicine (MD) or a Doctor of Osteopathic Medicine (DO) degree. Individuals who did not provide study consent were excluded. 

Participants arrived at the simulation suite in the Department of Anesthesia at times that fit their schedules. Each participant was provided with a written description of the study protocol and had the opportunity to ask questions. Additionally, all individuals were asked about their familiarity with all three intubation devices. If they were not familiar with a particular device, they were given time to practice single-lumen intubation on a separate manikin with the unfamiliar devices until they felt comfortable.

**Figure 1:**
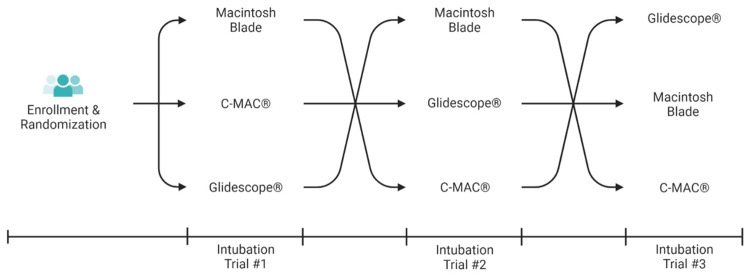
Three-way crossover study protocol. Each intubation trial was with a different laryngoscope, never repeating a previous laryngoscope. Each laryngoscope was used once for DLT intubation. DLT: double-lumen endotracheal tube.

**Figure 2:**
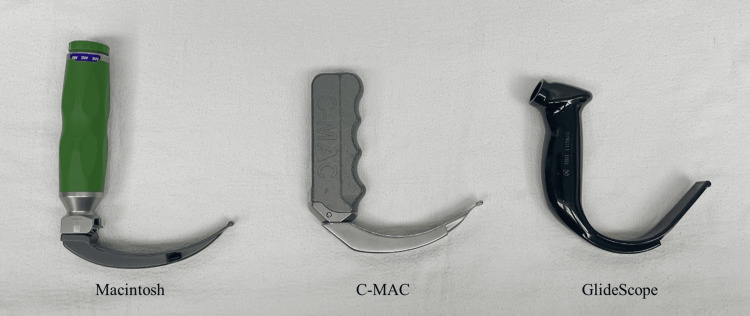
Picture of the laryngoscopes used. From left to right: Macintosh blade size 3, C-MAC® video laryngoscope with size 3 blade, and GlideScope® with a 60-degree angulated size 3 blade.

Once each participant felt comfortable with all three devices, they were randomized via a crossover design, with the order of devices determined using a Latin square design. There were six possible permutations for the order of devices used (Figure 1). The devices used included the C-MAC® video laryngoscope with a standard size 3 blade, the GlideScope® with a 60-degree angulated size 3 blade (Figure 2), and the Macintosh size 3 blade. Every participant intubated an adult manikin (Laerdal, Wappingers Falls, NY, USA), equipped with a head assembly and lungs, using each device, resulting in a total of three timed intubations. In the event of a failed intubation (esophageal intubation), participants were allowed to continue until they successfully intubated the trachea. For all three devices, the same Covidien Mallinckrodt left-sided 37-Fr DLT (Medtronic PLC, Dublin, Ireland) was used with its designated stylet that accompanied the tube. The participants were allowed to pre-shape the DLT using the stylet to match the configuration of the different types of blades as they wished.

Participants were allowed to set up the room at the simulation suite, such as positioning the device on the table and lubricating the DLT and its stylet, in a manner consistent with their own clinical practice in an operating room. They were instructed to place the DLT through the vocal cords; however, they did not need to achieve or confirm lung isolation. Once the participant believed that the DLT had passed through the vocal cords, the stylet was removed, and the tube was further advanced until resistance was felt. Participants then inflated a tracheal cuff on the DLT and connected the DLT to the Ambu® hyperinflation bag (Ambu Inc., Columbia, MD, USA). Successful intubation was confirmed when bilateral lungs were inflated by squeezing the Ambu® bag. Failed intubation was defined by having the DLT placed in the esophagus. This was confirmed by the failure to inflate the lungs when squeezing the Ambu® bag. This process was repeated with the other two devices. Participants were allowed to have an assistant help with removing the stylet, handing over the Ambu® bag, and applying cricoid pressure if necessary. The primary outcome measure was the time taken to achieve successful intubation, and the secondary outcome measure was the number of failed intubations. 

Data collection

Intubation time was measured from the first contact with either the DLT, intubation device, or manikin to the first sign of bilateral lung inflation upon squeezing the Ambu® bag. Time spent setting up the room at the simulation suite prior to the actual intubation was not counted as the intubation time. A stopwatch timer was used for measurement and was started/stopped based on visual inspection by a single evaluator. Failed intubation (esophageal intubation) was documented, and the subsequent successful intubations were included in the analysis. When intubation failed, the stopwatch timer was stopped, and then a new timer was started to measure the duration of the subsequent intubation attempt. Only the successful intubation times were included in the primary outcome analysis. The participants' postgraduate years of training were also recorded.

Data analysis

To evaluate the effectiveness of the different intubation devices, we formulated a null hypothesis for our statistical analysis. The null hypothesis posited that there would be no significant difference in the intubation time of the C-MAC® video laryngoscope, the GlideScope® video laryngoscope, and the Macintosh blade direct laryngoscope for endotracheal intubation using a DLT in an adult manikin. Our analysis aimed to test this null hypothesis, and we compared the meantime to intubation and the incidence of failed intubations across the three devices. Statistical analyses were performed using Excel’s Analysis ToolPak add-in (Microsoft Corp., Redmond, WA, USA). Mean, median, range, interquartile range, kurtosis, skewness, standard deviation, and standard error were obtained on the duration of successful intubation in all three devices. A Kruskal-Wallis one-way analysis of variance by rank was performed to assess for significance between time to intubation for all three devices and between time to intubation of each device by training level. A subsequent Mann-Whitney U test was used to locate significant differences between devices. Significance was determined by using an alpha set to 0.05. All data are presented as mean ± SD unless otherwise stated.

## Results

Ninety-four anesthesia providers enrolled in and completed the study. The participants were composed of six interns (postgraduate year 1, PGY-1), 49 first-year residents (PGY-2), three second-year residents (PGY-3), nine final-year residents (PGY-4), 15 anesthesia fellows (PGY-5 or more for specialized training), and 12 attending physicians (faculty).

**Figure 3:**
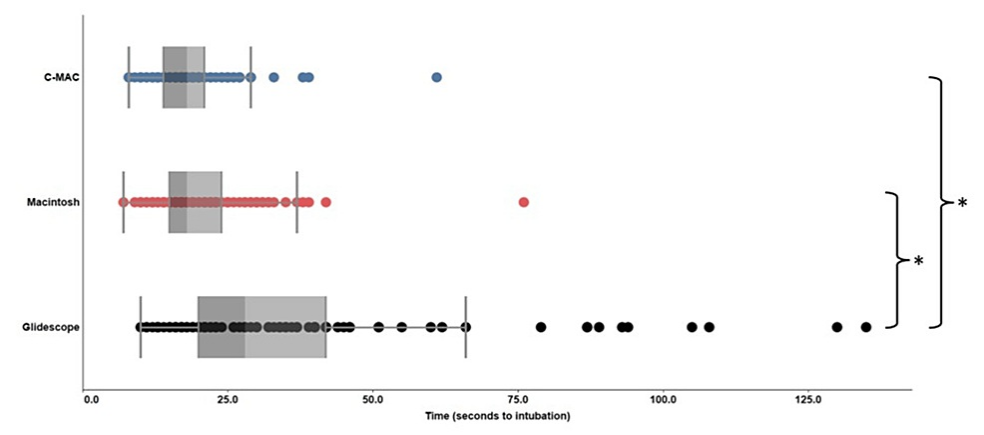
Comparison of the individual time to intubation for C-MAC® video laryngoscope versus Macintosh direct laryngoscope versus GlideScope® video laryngoscope. *P<0.001.

Each individual attempted intubation with three different devices, totaling 282 intubations. Times to intubation by the devices are illustrated in Figure 3. Mean times-to-intubation for the C-MAC®, GlideScope®, and Macintosh blades were 18.57 ± 0.77, 36.26 ± 2.69, and 20.76 ± 0.96 seconds, respectively. There was a significant difference (P<0.001) between the GlideScope® 60-degree angulated blade and the two other laryngoscope standard blades. No significant differences were detected between the C-MAC® and the Macintosh blades (P>0.05). Two individuals failed to place the DLT utilizing the Macintosh blade on their first attempt. The two failed attempts were by anesthesia trainees (PGY-3 and -4). Upon a second attempt, these individuals were able to place the DLT in the trachea rather than the esophagus. One of the successful intubations upon the second attempt is represented by the farthest most outlier data point in the Macintosh data set. The other subject who had a first-attempt failure was able to intubate within a non-outlier timeframe. The outlier data point in the C-MAC data set did not fail any of their first attempts.

Comparison of the average time to intubation for the C-MAC® standard blade versus the GlideScope® 60-degree angulated blade versus the Macintosh blade by year of training is shown in Table 1. Mean times to intubation with the C-MAC® and the Macintosh blades were shorter than with the GlideScope® 60-degree angled blade across all levels of training.

**Table 1:** Comparison of average intubation time (seconds) by year of training/practice. Values are presented as mean ± SD. PGY: postgraduate year. P values for the ANOVA test.

Subject classification	C-MAC	GlideScope	Macintosh	P-value
PGY-1	17.3 ± 4.5	29.0 ± 20.3	18.7 ± 5.3	0.021
PGY-2	15.9 ± 4.3	28.5 ± 22.1	19.0 ± 7.0	0.106
PGY-3	17.0 ± 8.3	24.3 ± 13.8	23.6 ± 19.5	0.288
PGY-4	14.3 ± 6.8	29.3 ± 15.4	19.7 ± 9.3	0.497
PGY-5	20.5 ± 8.1	43.0 ± 28.4	21.6 ± 7.4	0.002
Faculty	16.2 ± 6.1	36.5 ± 25.7	19.0 ± 7.5	0.755

## Discussion

In this manikin study, comparing three different laryngoscopes, we observed that the time required for successful DLT intubation was significantly shorter using both the Macintosh and C-MAC® standard blades compared to the GlideScope® 60-degree angulated blade. There was no significant difference in intubation time between the Macintosh and C-MAC® blades.

The curvature of the GlideScope® blade differs from that of the standard blades of the C-MAC® and the Macintosh. Specifically, the tip of the GlideScope® blade has a steeper 60-degree angulation compared to the approximately 30-degree angulation of the other two blades. In this study, using this blade with a 60-degree angle, anterior displacement of the tongue and chin was not often necessary during laryngoscopy. This was because the vocal cords were fully visualized on the monitor without much effort. However, the maneuver-creating anterior displacement of the tongue and chin-was essential for achieving optimal alignment of the oral, pharyngeal, and laryngeal axes for DLT intubation. Consequently, most participants initially positioned the DLT too far posteriorly relative to the epiglottis, resulting in a suboptimal trajectory toward the vocal cords. Although all participants eventually manipulated the DLT into the vocal cords, this resulted in a longer intubation time. Additionally, the unique 60-degree angulation of the GlideScope® blade, though advantageous for visualizing the vocal cords, may affect the time required for blade insertion into the mouth. These findings contrast with previous reports where single-lumen tube intubations were studied and the GlideScope® blade had a comparable intubation time to the Macintosh laryngoscope [12,13]. This discrepancy can be attributed to the DLT's larger diameter, its more cumbersome nature, and its reduced flexibility, especially when navigating the curve of the steeply angulated blade.

There are several studies investigating the effectiveness of various intubation devices for DLT intubation, yielding inconsistent results across different situations [14-18]. In most past studies, the operators performing the DLT intubation were either not specified or were experienced anesthesia providers, with many studies excluding anesthesia trainees. What distinguishes the current study is the inclusion of anesthesia trainees as operators. As a result, the mean time to successful DLT intubation was consistently the longest with the GlideScope® 60-degree angulated blade across all training levels, as shown in Table 1. This suggests that the inexperience in intubation skills could not have fully explained the differences in intubation time, and the results could be generalized regardless of the level of training.

Notably, of the 282 DLT intubations, two failed (resulting in esophageal intubation) on the first attempt, both using the Macintosh blade. The two failed intubation attempts were by anesthesia trainees (PGY-3 and -4). The fact that PGY-3 and PGY-4 residents were unable to intubate, unlike the PGY-1s, suggests that a lack of experience in intubation skills cannot fully explain the increased rate of failed intubations. This implies that the result could be generalized regardless of the level of training. However, with only two failed intubations out of 282, we do not have a sufficient number of cases to draw any definitive conclusion. While the current study did not ascertain whether participants had an adequate direct view of the vocal cords with the Macintosh laryngoscope, it is possible that inadequate visualization of the vocal cords contributed to the intubation failures. This observation was consistent with previous reports on single-lumen tubes, which revealed that the C-MAC® provided better glottic visualization compared to the direct laryngoscope [19,20], thereby reducing inadvertent esophageal intubations. Hence, the higher success rates and faster intubation times observed with the C-MAC® could hold significant clinical implications.

In the current study, an adult manikin with normal airway anatomy (Laerdal, Wappingers Falls, NY, USA) was used. This manikin has received a good rating as an airway training device, especially for DLT intubation [21], and was found to be a good alternative airway training device to fresh frozen cadaver models [22]. By using a manikin with consistent airway anatomy, we eliminated the patient’s airway anatomical variation as a variable. This allowed for more controlled experimentation, even with a small sample size when comparing the intubation devices. Subsequently, the results obtained from the current study using a manikin may help with the design of a future study to compare efficacy among intubation devices in a clinical setting.

There are several limitations to the current study. First, we used a 37 Fr left-sided DLT. Consequently, results might have varied if a different size of DLT had been used. Second, the manikin we used has normal airway anatomy; therefore, outcomes might differ in situations involving difficult airway management. Third, the assumption that the intubation experience increased in the postgraduate years could be inaccurate, as the prior experience of the participants before enrollment in the study with these devices and DLT was not recorded. Thus, it is acknowledged that variations in individual exposure to these specific devices and DLT intubation prior to the study could potentially influence outcomes. Lastly, successful intubation was defined by the placement of the DLT through the vocal cords and the subsequent ability to ventilate the lungs, and the positioning of the tip of the left-sided DLT in the left main bronchus was not confirmed either by fiberoptic bronchoscopy or by assessing one-lung ventilation. Therefore, the findings could be different if the primary outcome were defined as successful one-lung ventilation.

## Conclusions

The current study demonstrated that the C-MAC® and Macintosh standard size 3 blades are more efficient in time to a 37 Fr left-sided DLT placement in the manikin with normal airway anatomy compared to the GlideScope® 60-degree angulated size 3 blade. Intubation failures on the first attempt occurred with the Macintosh blade. These findings suggest that, of the three laryngoscopes studied, the C-MAC® is the most effective for timely and successful DLT intubation in a manikin with normal airway anatomy. Further studies are needed to confirm these results in human subjects.
